# Genetic moderation of the association between regulatory focus and reward responsiveness: a proof-of-concept study

**DOI:** 10.1186/2045-5380-3-3

**Published:** 2013-02-01

**Authors:** Elena L Goetz, Ahmad R Hariri, Diego A Pizzagalli, Timothy J Strauman

**Affiliations:** 1 Department of Psychology & Neuroscience, Duke University, Durham, NC, USA; 2 Institute for Genome Sciences and Policy, Duke University, Durham, NC, USA; 3 Department of Psychiatry, Harvard University Medical School, Boston, MA, USA

**Keywords:** Regulatory focus, Self-regulation, Reward responsiveness, Dopamine, *COMT*

## Abstract

**Background:**

Recent studies implicate individual differences in regulatory focus as contributing to self-regulatory dysfunction, particularly not responding to positive outcomes*.* How such individual differences emerge, however, is unclear. We conducted a proof-of-concept study to examine the moderating effects of genetically driven variation in dopamine signaling, a key modulator of neural reward circuits, on the association between regulatory focus and reward cue responsiveness.

**Method:**

Healthy Caucasians (*N*=59) completed a measure of chronic regulatory focus and a probabilistic reward task. A common functional genetic polymorphism impacting prefrontal dopamine signaling (*COMT* rs4680) was evaluated.

**Results:**

Response bias, the participants’ propensity to modulate behavior as a function of reward, was predicted by an interaction of regulatory focus and *COMT* genotype. Specifically, self-perceived success at achieving promotion goals predicted total response bias, but only for individuals with the *COMT* genotype (Val/Val) associated with relatively increased phasic dopamine signaling and cognitive flexibility.

**Conclusions:**

The combination of success in promotion goal pursuit and Val/Val genotype appears to facilitate responding to reward opportunities in the environment. This study is among the first to integrate an assessment of self-regulatory style with an examination of genetic variability that underlies responsiveness to positive outcomes in goal pursuit.

## Background

The concept of *self-regulation* describes a broad set of cognitive and behavioral processes by which individuals pursue their goals and respond dynamically to their perceived progress toward them [1]. There is increasing evidence that self-regulatory dysfunction constitutes a vulnerability factor for mood disorders and related forms of psychopathology [2,3]. Variability in goal pursuit strategies and the effectiveness of those strategies reflect differences in both top-down and bottom-up mechanisms as well as the characteristics of the interpersonal context in which goals are being pursued [4]. Although self-regulation has been studied at multiple levels of analysis, little research has examined how those mechanisms interact to influence people’s responsiveness to opportunities for goal attainment. Behavioral scientists are beginning to turn their attention to interactions between psychological mechanisms of self-regulation and neurobiological factors that underlie goal pursuit [5]. In this proof-of-concept study, we sought to examine the independent and interactive contributions of a psychological construct relevant to self-regulation of personal goal pursuit and a genetically-based neurobiological individual difference that affects reward-related cognitive processing. Specifically, we investigated whether individual differences in regulatory focus – a preference for pursuing desired end-states via strategic approach vs. avoidance, and/or variation in prefrontal dopamine signaling associated with *COMT* rs4680 genotype, predicted performance on a well-validated measure of reward responsiveness.

### Regulatory focus and goal pursuit

Regulatory focus theory (RFT) [6,7] proposes two cognitive/motivational systems for personal goal pursuit, the *promotion* and *prevention* systems. Both systems serve the purpose of pursuing positive end-states, but they differ in sensitivity to environmental cues as well as in goal pursuit means and strategies. The promotion system operates by approaching a match with the desired positive outcome and “making good things happen,” using eagerness as a means of pursuit. The prevention system operates by avoiding a mismatch with the positive outcome and “keeping bad things from happening,” using vigilance as a means of pursuit. RFT proposes that a history of success with one class of goal-pursuit strategies would be expected to induce a bias toward using those strategies, leading an individual to seek out contexts where preferred goals and strategies would be available [8]. RFT thus postulates stable individual differences in goal responsiveness based on the individual’s history of promotion and/or prevention socialization and goal pursuit experiences [9,10].

Individual differences in promotion and prevention orientation have dissociable neural correlates. For example, Eddington et al. [11] found that priming of individuals’ promotion (but not prevention) goals was associated with activation of the left orbitofrontal cortex (OFC), and magnitude of activation correlated with strength of orientation to promotion goal pursuit. Thus, relative preference for promotion goals is manifested in intensity of neural responses to personalized cues for such goals, suggesting that neurobiological differences could moderate the association between regulatory focus and sensitivity to cues for positive outcomes. Furthermore, chronic perceived failure to attain promotion goals is associated with dysphoric symptoms (e.g., [12]), and Eddington et al. [13] observed that individuals meeting DSM-IV criteria for major depressive disorder manifested attenuated left OFC activation in response to promotion goals. Thus, delineating the neurobiological mechanisms associated with self-regulatory dysfunction may provide new insights into the onset and maintenance of mood disorders [14].

### *COMT* genotype, dopaminergic signaling, and goal pursuit

Several lines of research have established the role of dopamine (DA) signaling in a mesocorticostriatal circuit to update reinforcement information, encode new reward contingencies, and facilitate incentive motivation [15,16]. DA signaling in the prefrontal cortex (PFC) is also important for working memory and attentional processes [17], underlying its role in goal pursuit. A common functional missense polymorphism (rs4680) in the gene for catechol-O-methyltransferase (COMT) – an enzyme responsible for the degradation of catecholamines including DA – is known to affect PFC synaptic DA availability [18-20] as well as relative DA signaling in the mesocorticostriatal circuit [21]. Specifically, the low-activity Methionine (Met) allele of rs4680 results in relatively higher tonic dopamine levels in the PFC, facilitating sustained DA firing and improved maintenance of activity states, as well as a higher threshold to switch activity states. In contrast, the Valine (Val) allele is associated with relatively decreased tonic PFC DA, and higher phasic activity in mesocorticostriatal circuits [21].

Several studies have probed moderating effects of rs4680 on mechanisms of self-regulation, including executive control, learning and adaptability, and affective processing. Both the Met and Val alleles are associated with improved performance and processing efficiency in different contexts, representing a *trade-off* in functionality [22,23]. Research on task switching and reversal learning has found that the Met/Met genotype is associated with increased cognitive stability while the Val/Val genotype is associated with increased cognitive flexibility [24,25]. Thus, the Met allele is associated with improved executive cognition, more efficient PFC function [26], increased attentional control (e.g., [27]), as well as reward-related activation in the PFC [28,29]. In contrast, the Val allele is associated with better adaptability to new contingencies and efficient shifting between informational states, allowing for better reversal learning and serving a protective function against stress and negative affect in the face of failure (e.g., [30,31]). However, the association between rs4680 genotype and reward responding is not straightforward: Val allele carriers have been found to better differentiate win and loss experiences, which can facilitate learning (e.g., [32]), while Met allele carriers have been shown to have potentiated responsiveness to rewards due to higher DA availability (e.g., [29,33]).

Given the importance of task parameters and motivational context in determining which allele is advantageous for regulating goal pursuit, a main effect of rs4680 genotype is unlikely. There is no particular rs4680 genotype that is uniformly advantageous across circumstances and no categorical “risk” genotype; instead, the particular mechanisms, tendencies, and vulnerabilities associated with each genotype are best considered as a trade-off where the outcome depends on the contingencies in the environment. Both proximal and long-term environmental conditions interact with genetic variability to produce particular context-specific advantages and disadvantages in task performance, behavior, and affect.

A similar cost/benefit framework can be found in psychological theories of self-regulation, such as RFT. For example, individuals differ in their capacity to self-regulate and have different styles and strategies of pursuing their goals. They also differ in their responsiveness to feedback about their strategies and their resilience in the face of set-backs. These individual differences may prove to be adaptive or maladaptive depending on the circumstances in which goal pursuit occurs.

Given the commonality that psychological mechanisms of self-regulation and underlying behavioral genetics both frequently involve trade-offs, it is essential to integrate behavioral theories of individual differences in goal pursuit with knowledge about underlying genetic variability. In particular, these factors are likely to interact to produce responses to reward not predictable from either mechanism alone, and potentially constitute pathways to disordered behavior and psychopathology. The present study examined whether the interaction between rs4680 genotype and individual differences in regulatory focus predicted responses to cues for reward.

### Modeling the interactive effects of regulatory focus and *COMT* genotype

To measure reward responsiveness, we selected a well-validated laboratory probabilistic reward task designed to provide an experimentally-derived operationalization of reward responsiveness and learning. This task has been used to assess reward responsiveness in healthy populations [34], individuals under stress [35,36], and individuals with depression [37]. In this task, reward responsiveness is operationalized as the tendency to preferentially select a more frequently rewarded stimulus and thus to develop a response bias.

The task offers several advantages as an index of responsiveness to cues for goal attainment. First, it has been characterized as a signal detection task (e.g., [34]), and the signal detection framework has been previously applied to describe the promotion and prevention systems [38,39]. Individuals in a promotion focus seek to achieve “hits” and ensure against errors of omission, resulting in a bias of saying “yes” to a cue that might signify an opportunity for reward. Conversely, individuals in a prevention focus are concerned with making correct rejections and ensuring against errors of commission, resulting in a bias of saying “no” to a potential reward and avoiding incorrect responses. This task in particular is promotion-consistent because the goal is to earn money (rather than to “avoid losing” money) and the payoff structure is framed as gain versus non-gain. Since promotion focus entails a preference for opportunities to make good things happen and a relative attention to cues for reward as well as increased use of approach strategies, the task is a “fit” [7] for a chronic orientation toward promotion goals. Second, the task has been shown to measure reward-related cognitive processes directly relevant to self-regulation of goal pursuit, such as responding to positive feedback and capitalizing on opportunities for gain [34]. Finally, the task provides an efficient way to quantify goal-directed behavior, namely, an accumulation of trial-by-trial responding in pursuit of monetary rewards.

We hypothesized that individual differences in promotion system strength would predict reward-related behavior in our experimental task. However, we also hypothesized that DA signaling in the PFC as modeled by rs4680 genotype would moderate the association between promotion goal pursuit orientation and reward learning. Specifically, we predicted that the rs4680 Val allele would be associated with greater behavioral modulation and flexibility in reward-responsive behavior for individuals with different experiences of promotion goal pursuit. On the other hand, we predicted that the rs4680 Met allele would be associated with a more stable profile of reward responsiveness, regardless of previous goal pursuit experiences or promotion system strength.

## Method

### Participants

To minimize possible population stratification due to different rs4680 allele frequencies across ethnic groups, our study sample was restricted to Caucasian participants. Study participants (*N*=67) were recruited via on-campus flyers. All participants were healthy volunteers between 18 and 30 years of age and were self-reported non-smokers, due to previous reports that nicotine has significant effects on reward responsiveness as assessed by the current probabilistic reward task [40]. Participants provided written informed consent, were compensated $10 per hour, and earned a pre-determined amount between $5.80 and $6.20 in the computer task.

Four participants were excluded for non-compliance (*N*=3) or computer malfunction (*N*=1). One subject was excluded due to high levels of depressive symptoms, and three subjects were excluded from analyses due to outlier scores (see Data Analysis section for details). Thus, complete and valid data were available for *N*=59 subjects (35 female). The participants had a mean age = 21.3 ± 2.7 and 92% were right-handed as per self-report. The participants were generally free of psychological distress (mean Beck Depression Inventory-II (BDI-II) score = 5.93, SD = 4.86, range = 1 to 18), although information about medication use and psychiatric history was not collected.

### Procedures

Participants completed the 20-min probabilistic reward task and a set of self-report questionnaires, including the Regulatory Focus Questionnaire (RFQ) [8], the BDI-II [41], and the trait version of the Positive and Negative Affect Schedule (PANAS) [42]. In addition, participants gave a saliva sample for genetic analysis. All procedures were approved by the Duke University Institutional Review Board for Non-Medical Research.

#### Probabilistic reward task

This computerized task is a probabilistic reward-based learning paradigm where participants are asked to identify which of two stimulus images is being presented on each trial. This task has been described in detail elsewhere (e.g., [34]) and has been adopted from Tripp and Alsop [43]. Briefly, participants are instructed press a button on the keyboard to indicate whether a long (13 mm) or short (11.5 mm) mouth is presented (100 ms) within a schematic face. They are told that for *some* of their correct responses, they will receive a monetary reward of 5 cents. One of the images is rewarded more frequently, with a 3:1 reward ratio between the “rich” stimulus and the “lean” stimulus. The task consists of three blocks of 100 trials each, with up to 40 trials per block receiving a reward. The two mouth types are presented with equal frequency, but, unknown to the participants, the reward feedback is asymmetrical in favor of the “rich” stimulus (30 rich versus 10 lean rewards). This paradigm has been found to reliably produce a response bias such that as the task proceeds, the “rich” or more frequently rewarded stimulus is preferentially selected [34,36].

Response bias and discriminability scores were calculated according to the following formulae [34]:

$$\text{log}b=\frac{1}{2}\text{log}\left(\frac{Rich_{\text{correct}}\;*\;Lean_{\text{incorrect}}}{Rich_{\text{incorrect}}\;*\;Lean_{\text{correct}}}\right)$$

$$\text{log}d=\frac{1}{2}\text{log}\left(\frac{Long_{\text{correct}}\;*\;Short_{\text{correct}}}{Long_{\text{incorrect}}\;*\;Short_{\text{incorrect}}}\right)$$

Both formulae were adjusted by adding 0.5 to each value of correct or incorrect responses to eliminate zero values in the denominator (see [37]). Discriminability scores were used to ensure that any findings observed for response bias were not artifacts of task difficulty or individual variability in skill. Response bias towards the more frequently rewarded stimulus can be interpreted as the degree to which an individual engages in the task based on her/his reinforcement history, and as an index of the capacity to respond to reward information and maintain the bias even when reinforcement is intermittent [34,43]. A high response bias results if a participant has a high hit rate for the rich stimulus and a high miss rate for the lean stimulus, reflecting a strategy where participants try to ensure “hits” where they are rewarded for their response (gains), and to ensure against errors of omission (non-gains).

To capture participants’ propensity to modulate behavior as a function of prior reward, total response bias across the three blocks of the task was used as the outcome variable. Bias and discriminability were calculated across all valid trials, defined as one where the response reaction time (RT) was between 150 and 2500 msec, and the natural log transformation of RT was within 3 standard deviations of the mean for each participant, as in previous studies using this task (e.g., [34]). A total of 1.43% of the trials were excluded.

#### Individual differences in regulatory focus

The Regulatory Focus Questionnaire (RFQ) [8] is a 22-item, self-report Likert-style measure designed to assess individual differences in orientation toward promotion and prevention. The questionnaire has four subscales, two that measure individuals’ recollection of their early experiences in being oriented toward a promotion or prevention focus by their parents (“history” subscales), and two that measure individuals’ subjective assessment of their experiences achieving goals of either a promotion or prevention nature (“success” subscales). Sample items include “My parents encouraged me to try new things” (promotion history), “My parents kept order in our house by having a lot of rules and regulations for me” (prevention history), “I feel like I have made progress towards being successful in life” (promotion success), and “Not being careful enough has gotten me into trouble at times” (prevention success, reverse-scored). Responses are made on a 5-point scale ranging from “never or seldom” or “certainly false” to “very often” or “certainly true,” and the item responses within each subscale are averaged to produce four scores: promotion history, prevention history, promotion success, and prevention success. Higgins et al. [8] reported good internal reliability for the promotion success scale (α = 0.73) and prevention success scale (α = 0.80), and the scales showed a two month test-retest reliability of 0.79 correlation or higher. There are no published psychometric data for the history scales, although in the current sample, the reliability scores for the history subscales were α = 0.70 for promotion history, and α = 0.80 for prevention history.

#### Genotyping

Saliva samples were collected via Oragene kits (Oragene, DNA Genotek; Ottawa, Ontario, Canada). The samples were purified and DNA was extracted and rehydrated according to standard protocols (http://www.dnagenotek.com). Genotyping of rs4680 was performed using TaqMan allele-specific polymerase chain reaction (PCR) as per Caspi et al. [44]. All genotype calls were ascertained by two independent raters using sequence verified standards with 100% agreement.

#### Data analysis

Extreme response bias scores (>3 standard deviations from the mean) of two participants were removed to improve the normality of the data. One participant had two RFQ subscale scores that were >3 standard deviations from the mean and these exerted moderate to high influence on the association of those subscale scores and response bias scores. To prevent statistical distortion due to these outlier scores, this participant was also removed from the final analyses. Genotypes were coded as the number of Met alleles for rs4680.

## Results

### Distribution of genotypes in the sample

The sample was split into three groups on the basis of genotype: Met/Met (*N*=14, 24% of sample), Val/Met (*N*=28, 47%), and Val/Val (*N*=17, 29%). The three groups were in Hardy-Weinberg equilibrium (*χ*^2^(2)=.14, *p*=.93). The Pearson correlations among the RFQ scores, PANAS trait scores, and response bias are displayed in Table 1.

**Table 1:** Correlation matrix for study variables

	**Promotion history**	**Prevention success**	**Promotion success**	**PANAS PA**	**PANAS NA**	**Total RB**
**Prevention History**	0.05	-.27*	-.34**	-.11	-.33*	-.13
	*p*>.1	*p*=.036	*p*=.008	*p*>.1	*p*=.012	*p*>.1
**Promotion History**		.38**	.15	.27*	-.26*	-.07
		*p*=.003	*p*>.1	*p*=.038	*p*=.048	*p*>.1
**Prevention Success**			.74**	.11	-.04	.13
			*p*<.001	*p*>.1	*p*>.1	*p*>.1
**Promotion Success**				.15	-.06	.14
				*p*>.1	*p*>.1	*p*>.1
**PANAS PA**					.04	-.14
					*p*>.1	*p*>.1
**PANAS NA**						.19
						*p*>.1
**Total RB**						1

* *p*<.05, ** *p*<.01; PANAS = Positive and Negative Affect Schedule, PA = Positive Affect, NA = Negative Affect, RB = Response Bias.

### Discriminability and accuracy

A set of regression analyses examined whether any of the RFQ variables or genotypes predicted total discriminability or accuracy scores as main effects or interactions, and all models and interaction effects were non-significant (all *p* > .1).

### Regulatory focus and genotype predicting response bias

We conducted a series of analyses to determine whether individual differences in regulatory focus and/or *COMT* rs4680 genotype predicted total response bias on the reward task. First, a one-way ANOVA was performed to examine whether response bias differed by genotype group; this test was not significant (*F*(2, 56) = 0.52, *p* > .5). In addition, ANOVAs were performed to examine whether any of the regulatory focus variables differed by genotype group, using a Bonferroni correction to control for Type 1 error (4 tests, α threshold =.0125). Only prevention history was found to differ by rs4680 group, *F*(2,56) = 4.73, *p* < .05. A post-hoc Tukey pairwise comparison test revealed that the rs4680 Val/Val group had significantly higher prevention history scores than the Val/Met group (group means = 4.17 versus 3.56 respectively, *p* < .05) but not the Met/Met group (group mean = 4.11, *p* > .9). See Table 2 for descriptive statistics of key study variables by *COMT* rs4680 genotype group.

**Table 2:** **Descriptive statistics (means and standard deviations) for study variables by *COMT *genotype group**

***COMT*group**	**Prevention history**	**Promotion history**	**Prevention success**	**Promotion success**	**BDI-II**	**PANAS PA**	**PANAS NA**	**Total RB**
**Val/Val**	4.17 (0.58)	3.81 (0.93)	2.92 (0.99)	3.29 (0.86)	5.00 (3.94)	32.06 (6.16)	13.41 (4.17)	0.11 (0.15)
**Val/Met**	3.56 (0.78)	3.99 (0.68)	2.93(1.04)	3.39 (0.81)	6.93 (5.13)	34.46 (5.47)	14.29 (3.79)	0.11 (0.14)
**Met/Met**	4.11 (0.78)	4.13 (0.59)	2.87(1.15)	3.25 (1.14)	5.07 (5.24)	32.50 (6.43)	13.57 (3.80)	0.16 (0.18)

PANAS = Positive and Negative Affect Schedule, PA = Positive Affect, NA = Negative Affect, RB = Response Bias.

Next, hierarchical regression analysis was used to test the prediction that regulatory focus and rs4680 genotype interacted to predict response bias. In the first step, the four RFQ variables were entered. This step was non-significant, *F*(4, 54) = .56, *p* = .69, and all βs were non-significant (all *p* > .4). In the second step, rs4680 genotype was added to the model but did not significantly improve fit, Δ*F*(1, 53) = 1.04, *p* = .31. For the third step, the RFQ variables were mean-centered and regulatory focus X rs4680 interaction terms were created. Each interaction term was entered into the model to evaluate its unique contribution above the main effects of all RFQ variables and the main effect of rs4680. The interaction terms for prevention history, promotion history, and prevention success did not provide significant incremental improvement in model fit: all Δ*F p* > .60. However, the promotion success X rs4680 interaction was a significant predictor of response bias: Δ*F*(1, 52) = 4.13, *p* < .05, ΔR^2^ = 0.07 (see Additional file 1: Table S1). In a reduced model where only promotion success, rs4680 genotype, and the interaction were entered, the overall model fit was marginally significant: *F*(3, 55) = 2.39, *p* < .08, R^2^ = 0.12.

**Figure 1:**
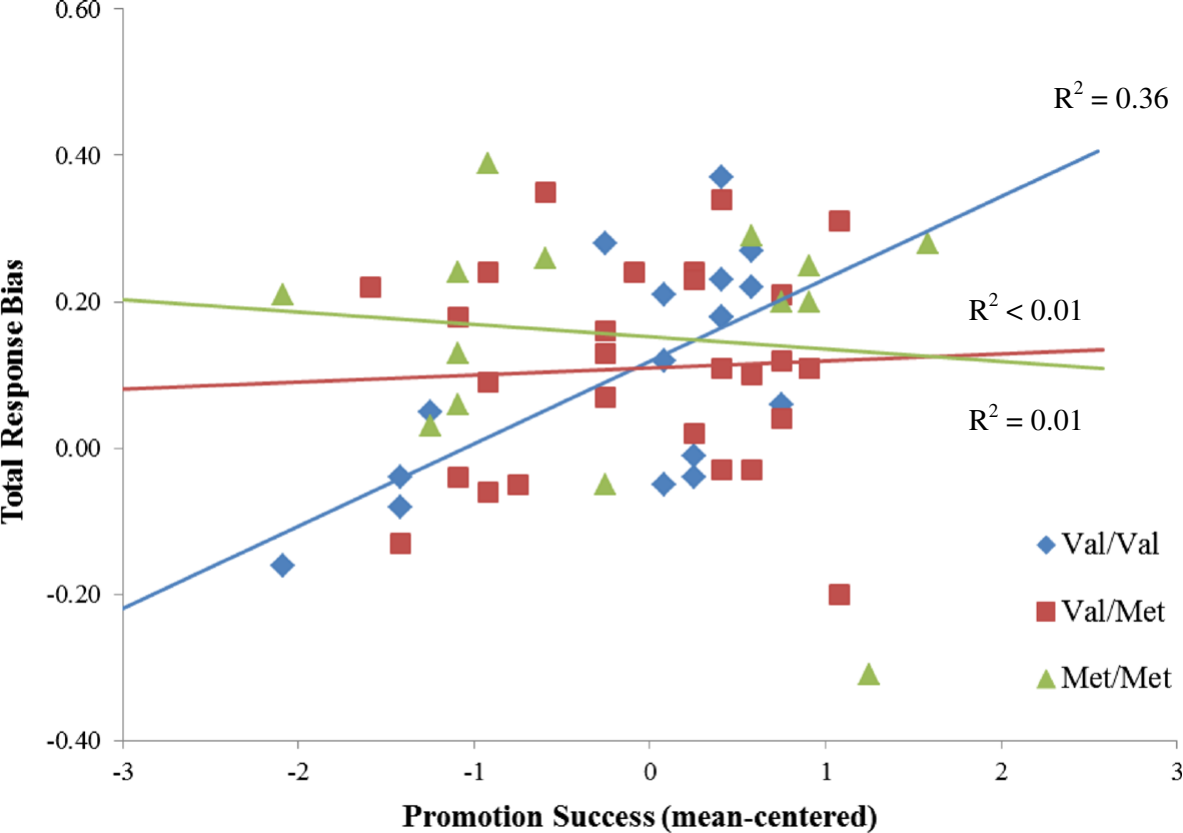
**Total response bias as a function of promotion success score, across *COMT *rs4680 genotype groups.** Promotion success predicted response bias only for the Val/Val participants, *p* < .01, adjusted R^2^ = .36.

Next, the promotion success X rs4680 interaction was decomposed by dividing the sample into genotype groups and examining the linear association between promotion success and response bias within each group. These regression analyses revealed that a significant association between promotion success and response bias was present only in the Val/Val genotype group: *F*(1, 15) = 9.85, *p* < .01, adjusted R^2^ = .36. The Val/Met and Met/Met groups did not manifest any association between promotion success and response bias (both *F* < .15; see Figure 1).

Finally, a tertiary split was performed on promotion success scores in order to compare mean response bias across the range of promotion success. The *COMT* rs4680 genotype groups Val/Met and Met/Met were combined. Total response bias was significantly lower for the Val/Val group compared to Met allele carriers in the lowest third of promotion scores, t(18) = −2.30, *p* = .034 (Additional file 2: Figure S1). There were no significant differences in response bias across genotype groups between those participants in the middle (*p* > .82) or highest third (*p* > .50) of promotion success.

## Discussion

The goal of this proof-of-concept study was to examine how regulatory focus and a common functional polymorphism affecting DA signaling predicted behavior in a probabilistic reward task. The results confirmed our hypothesis that rs4680 genotype, which biases COMT function and associated DA signaling in the PFC, moderates the degree to which a particular pattern of self-regulatory success experiences affects reward responsiveness. Specifically, our results showed that the combination of success in promotion goal pursuit and Val/Val genotype appears to facilitate responding to reward opportunities in the environment. On the other hand, Met allele carriers did not show an association between regulatory success in pursuing promotion goals and developing a reward-related response bias in our task.

Research examining the nature and consequences of self-regulation may provide a context for interpreting the present findings, both from the perspective of basic science and in regard to potential pathways of vulnerability for mood disorders. For example, we suggest that it is important to recognize that the observed associations among regulatory focus, rs4680 genotype, and reward responsiveness are not readily apparent at the phenotypic level. There was no broad association found between rs4680 genotype and self-regulatory style. This finding is consistent with previous research indicating that regulatory orientation is primarily shaped by accumulated experience with success or failure in goal pursuit and is independent from biological factors such as temperament [9], and also with the fact that variability in both mechanisms involves trade-offs that manifest in context-specific ways.

In this study, we hypothesized that an interaction of rs4680 genotype and self-regulatory style would result in predictable effects on responses to probabilistic reward feedback. The reward task yielded a reliably quantifiable measure of reward responsiveness, that is, participants’ propensity to modulate behavior as a function of prior reward. Individuals who are more responsive to reward feedback will preferentially select the more frequently rewarded stimulus and thus manifest a stronger response bias across the task. As noted previously, the task is promotion-consistent because outcomes are presented to participants as gain/non-gain – to earn money (instead of to “avoid losing” money). Therefore, individuals who believe that they are successful at achieving promotion goals should manifest a response bias that favors the more rewarded or “rich” stimulus. Interestingly, we did not observe a main effect of rs4680 genotype or promotion orientation on reward responsiveness. Rather, the effect of a history of promotion success on reward responsiveness was found to be moderated by rs4680 genotype (specific to the Val/Val genotype group).

The pattern of findings revealed a terminative interaction, where among individuals reporting relatively low levels of success pursuing promotion goals, the Val/Val genotype was associated with significantly lower response bias than in Met allele carriers. However, among individuals reporting frequent success experiences in the promotion domain, the Val/Val genotype was associated with mean response bias that did not significantly differ than that observed for Val/Met or Met/Met genotypes. Self-reported promotion success did not predict response bias among Val/Met or Met/Met genotypes, which also did not differ from each either. Thus, no Met allele dose effect was observed either as a main effect or as an interaction.

This pattern of results corresponds with the trade-off framework for interpreting *COMT*-related variability in reward sensitivity that was described above. The Val/Val individuals who reported low levels of promotion success demonstrated significantly lower total response bias scores than Met allele carriers. There is the possibility that Val/Val individuals are more susceptible to fluctuations in DA when rewards are omitted and therefore have greater difficulty integrating reward-related information over time. This would be an example of flexibility and behavioral adaptability being disadvantageous within a particular context, and such a neurobiological profile would be especially maladaptive given a personal history of low promotion success. Thus, Val/Val individuals with low promotion success might constitute a group of individuals who would be particularly vulnerable to anhedonia or reward insensitivity in the face of chronic promotion failure experiences. There is evidence that Val/Val individuals show decreased responsiveness to reinforcers [28,29], and reduced motivational drive to pursue reinforcers would interfere with successful promotion goal pursuit.

The fact that Met allele carriers did not show modulation of their response bias based on their degree of success in pursuing their promotion goals fits with a “resilience” model. Even in the context of low promotion success, individuals with higher tonic PFC dopamine (Met allele carriers) did not manifest low levels of reward-responsive behavior. However, there is a competing interpretation: that Met allele carriers are not able to increase reward-related responding based on their previous goal-pursuit experiences, which might adversely affect the pursuit of promotion goals. Such an alternative hypothesis is consistent with previous findings of cognitive [24,25] and affective [30] inflexibility associated with the Met allele. There may be some relative deficits in reward-related responding for Met allele carriers who have had successful self-regulatory experiences, because those individuals with high promotion success scores exhibited only average response bias scores. The Met allele carriers might have an inability to update or adapt behavior via reward feedback that could create some vulnerability in the face of future self-regulatory challenges or failure experiences.

The results from this study illustrate that an integrative approach to *COMT* variability and self-regulation has the potential to reveal novel pathways of psychological vulnerability. Self-regulation involves a complex set of processes including goal selection and pursuit, cognitive and emotional control, and ongoing decision-making [45]. Factors such as reward responsiveness, impulsivity/distractibility, and affective resilience can impact how goals are pursued and how feedback about progress is managed, and individual variability in *COMT* has been shown to affect all of these processes. A research strategy that applies individual differences information from genetic sources to studies of ongoing, situationally-embedded self-regulation might yield context-sensitive examples of when these processes break down and produce regulatory dysfunction and, possibly, clinical disorders.

Regions in the PFC, particularly the OFC, are involved in responsiveness to motivationally salient feedback and shifts in goal pursuit originating from bottom-up processes (e.g., [28]) as well as top-down processes (e.g., [11]). Thus, the OFC is likely to be a site that mediates the interaction between individual differences in regulatory focus and rs4680 genotype to produce reward-related behavior, and at least one study [13] has observed that clinically depressed individuals manifest attenuated left OFC activation in response to their own promotion goals.

The significant interaction between promotion success and rs4680 genotype was detected even in the hierarchical multiple regression models that included other regulatory focus variables as covariates. These analyses were particularly important to differentiate promotion success from prevention success, which were substantially correlated (*r*=.74, *p*<.001). Despite the intercorrelation, it was success in attaining promotion goals, and not successful prevention goal attainment, that predicted reward responsiveness for the Val/Val genotype. RFT postulates fundamental differences between the promotion and prevention systems with regard to the targets of goal pursuit (e.g., “ideals” vs. “oughts”), the strategies used to pursue them (“making good things happen” vs. “keeping bad things from happening”), and the motivational impetus that underlies goal pursuit (eagerness vs. vigilance). These behavioral and cognitive distinctions appear to be accompanied by differences in cortical activation when promotion vs. prevention goals are primed [11,13], although additional research is needed to characterize the shared and unique neural circuitry associated with these two hypothetical cognitive/motivational systems. Such research, in combination with a genetic/neurobiological level of analysis, could help to elucidate pathways by which self-regulatory dysfunction could lead to the onset and maintenance of mood disorders.

There are several limitations of this study that should be acknowledged. First, the relatively small sample size leaves us vulnerable to statistical noise including false positives. However, this is more problematic in the absence of significant effects (i.e., false negatives), which could simply reflect inadequate power, and thus one should use caution when interpreting non-significant results such as the lack of main effects. The fact that we found significant effects robust to the influence of covariates suggests the observed interaction between self-regulatory style and rs4680 genotype is valid. Nevertheless, replication of the effects described herein is necessary to further the possible utility of our interaction model for understanding clinically relevant outcomes (e.g., risk for mood disorders). Second, because the genetic analyses were restricted to a Caucasian-only sample, generalizability may be limited. It will be of interest to examine this effect in non-Caucasian samples. Third, although the task provided a well-validated operationalization of the experience of responding to reward feedback, its external validity as a proxy of goal pursuit behavior is understandably limited. Previous studies of the neurobiological substrates underlying regulatory focus relied on idiographic stimuli that captured individuals’ personal goals [11,13], and the degree to which earning money was a goal of the participants in this study was implied by their participation but not explicitly verified. A future study could extend these findings by obtaining genetic information on participants who undergo neuroimaging when exposed to their goals, or using another kind of goal-pursuit task such as anagram solving that is related to perseverance and conscious decision-making about effort and motivation. In addition, a future exploration of these effects should include measures of potential important contributory variables such as stressful life events (particularly during development) and state-level anxiety, as these both could impact the development and manifestation of one’s regulatory focus.

## Conclusions

This study used a research strategy that integrated neurobiological variability (as indexed by a common functional genetic polymorphism) and trait-like differences in cognitive/motivational systems to predict reward-related behavior in a novel way. Our findings showed that – consistent with a trade-off model – rs4680 genotype interacts with self-regulatory success experiences to predict reward responsive behavior only in individuals with a Val/Val genotype, and this pattern could be adaptive at high levels of success or maladaptive at low levels of success. By contrast, the response profiles of Met allele carriers indicated that previous goal pursuit success does not impact their development of a reward-related response bias. Our approach extends our understanding of how top-down self-regulatory mechanisms affect behavior by examining the moderating influence of bottom-up biological mechanisms. This interaction of mechanisms and methodologies is likely to be a fruitful avenue of future inquiry to elaborate the individual differences affecting complex behaviors and psychiatric phenotypes, such as reward dysfunction in depression or addiction [46].

## Abbreviations

(RFT): Regulatory focus theory; (COMT): Catechol-o-methyltransferase; (DA): Dopamine; (RFQ): Regulatory Focus Questionnaire.

## Competing interests

DAP has received consulting fees from ANT North America Inc. (Advanced Neuro Technology), AstraZeneca, Shire, and Ono PharmaUSA, and honoraria from AstraZeneca for studies unrelated to this project. The authors declare that they have no other competing interests.

## Authors’ contributions

ELG helped conceive of the design of the study, collected data and conducted genotyping, performed data analysis, and took the lead on writing the manuscript. ARH helped conceive of the design of the study, oversaw genetic analysis, and contributed to writing the manuscript. DAP contributed to the design of the study and data interpretation, as well as consulted on the implementation of the experimental task and provided feedback on the manuscript. TJS helped conceive of the design of the study, contributed to data analysis, and contributed to writing the manuscript. All authors read and approved the final manuscript.

## Supplementary Material

Additional file 1: Table S1 Regression results: Regulatory focus variables, *COMT* rs4680 genotype, and interactions predicting total response bias. Click here for file

Additional file 2: Figure S1 *COMT* rs4680 Val/Val verses Met-carrier response bias scores by promotion success groups. The * indicates that mean response bias values were significantly different, *p*<.05. Click here for file
