# Supplementary material for: Genetic moderation of the association between regulatory focus and reward responsiveness: a proof-of-concept study
Source: Biol Mood Anxiety Disord. 2013 Feb 1;3:3. doi: 10.1186/2045-5380-3-3 (PMC3570330; doi:10.1186/2045-5380-3-3)
Supplement: Additional file 1: Table S1 — Regression results: Regulatory focus variables, COMT rs4680 genotype, and interactions predicting total response bias. [file 2045-5380-3-3-S1.doc]

*Table 1s*

Regression results: Regulatory focus variables, *COMT* rs4680 genotype, and interactions predicting total response bias

Promotion Success models

| **Step** | **Predictor** | **Standardized β** (final model) | **Significance** | **Model R^2^** (includes all above predictors) | **ΔR^2^** | **ΔR^2^ significance** |
| --- | --- | --- | --- | --- | --- | --- |
| 1 | Prevention History | -0.09 | 0.53 |  |  |  |
|  | Promotion History | -.05 | 0.76 |  |  |  |
|  | Prevention Success | 0.11 | 0.62 |  |  |  |
|  | Promotion Success | 0.10 | 0.44 | 0.04 | 0.04 | 0.69 |
| 2 | rs4680 Genotype | 0.44 | 0.12 | 0.06 | 0.02 | 0.31 |
| 3 | Promotion Success X rs4680 | -0.49 | 0.05 | 0.13 | 0.07 | 0.05 |

Prevention Success models

| **Step** | **Predictor** | **Standardized β** (final model) | **Significance** | **Model R^2^** (includes all above predictors) | **ΔR^2^** | **ΔR^2^ significance** |
| --- | --- | --- | --- | --- | --- | --- |
| 1 | Prevention History | -0.07 | 0.66 |  |  |  |
|  | Promotion History | -0.15 | 0.34 |  |  |  |
|  | Prevention Success | 0.19 | 0.48 |  |  |  |
|  | Promotion Success | 0.05 | 0.81 | 0.04 | 0.04 | 0.69 |
| 2 | rs4680 Genotype | 0.14 | 0.32 | 0.06 | 0.02 | 0.31 |
| 3 | Prevention Success X rs4680 | -0.08 | 0.76 | 0.06 | <0.01 | 0.76 |

Promotion History models

| **Step** | **Predictor** | **Standardized β** (final model) | **Significance** | **Model R^2^** (includes all above predictors) | **ΔR^2^** | **ΔR^2^ significance** |
| --- | --- | --- | --- | --- | --- | --- |
| 1 | Prevention History | -0.05 | 0.72 |  |  |  |
|  | Promotion History | -0.13 | 0.55 |  |  |  |
|  | Prevention Success | 0.14 | 0.54 |  |  |  |
|  | Promotion Success | 0.06 | 0.81 | 0.04 | 0.04 | 0.69 |
| 2 | rs4680 Genotype | 0.14 | 0.31 | 0.06 | 0.02 | 0.31 |
| 3 | Promotion History X rs4680 | -0.04 | 0.87 | 0.06 | <0.01 | 0.87 |

Prevention History models

| **Step** | **Predictor** | **Standardized β** (final model) | **Significance** | **Model R^2^** (includes all above predictors) | **ΔR^2^** | **ΔR^2^ significance** |
| --- | --- | --- | --- | --- | --- | --- |
| 1 | Prevention History | -0.17 | 0.54 |  |  |  |
|  | Promotion History | -0.15 | 0.32 |  |  |  |
|  | Prevention Success | 0.17 | 0.46 |  |  |  |
|  | Promotion Success | 0.03 | 0.89 | 0.04 | 0.04 | 0.69 |
| 2 | rs4680 Genotype | 0.11 | 0.46 | 0.06 | 0.02 | 0.31 |
| 3 | Prevention History X rs4680 | 0.14 | 0.62 | 0.06 | <0.01 | 0.62 |
