# Supplementary material for: Genetic moderation of the association between regulatory focus and reward responsiveness: a proof-of-concept study
Source: Biol Mood Anxiety Disord. 2013 Feb 1;3:3. doi: 10.1186/2045-5380-3-3 (PMC3570330; doi:10.1186/2045-5380-3-3)
Supplement: Additional file 2: Figure S1 — COMT rs4680 Val/Val verses Met-carrier response bias scores by promotion success groups. The * indicates that mean response bias values were significantly different, p<.05. [file 2045-5380-3-3-S2.docx]

*Figure 1s*. *COMT* rs4680 Val/Val verses Met-carrier response bias scores by promotion success groups. The * indicates that mean response bias values were significantly different, *p*<.05.

*
